# PEGylated porcine–human recombinant uricase: A novel fusion protein with improved efficacy and safety for the treatment of hyperuricemia and renal complications

**DOI:** 10.1515/biol-2022-0799

**Published:** 2024-01-23

**Authors:** Xiangyan Wang, Hao Lu, Jun Rong, Zhongjie Sun, Yanhua Zheng, Bolin Fan, Ziming Jia

**Affiliations:** Hubei Provincial Key Laboratory for Applied Toxicology, Hubei Provincial Center for Disease Control and Prevention, Wuhan 430079, China; College of Life Science, Yangtze University, Jingzhou 434000, PR China; Jing Zhou Chang Xin Biotechnology Co., Ltd, Jingzhou 434000, PR China

**Keywords:** urate oxidase, protein expression and purification, PEGylation, preclinical test

## Abstract

The growing prevalence of hyperuricemia necessitates the urgent development of more potent treatments. This study aimed to develop, optimize, and evaluate the safety and efficacy of porcine–human recombinant uricase (PHRU) both *in vitro* and *in vivo*. The study employed gene editing of PHRU through site-directed mutagenesis, with recombinant proteins expressed *in vitro* utilizing *Escherichia coli*. The polyethylene glycol (PEG) approach was employed to augment uricase stability and diminish immunogenicity. The pharmacokinetics and pharmacodynamics of PHRU were tested *in vitro* and in Sprague Dawley rats. Successful expression of the fusion protein in *E. coli* and the development of the PEGylated drug were achieved. *In vitro* experiments confirmed the efficacy of PEG-PHRU in degrading uric acid, with PEGylation not markedly affecting the biological activity of PHRU. Animal studies revealed that PEG-PHRU significantly lowered plasma uric acid levels and mitigated hyperuricemia-induced renal damage in rats. Both drug metabolism and pharmacokinetics exhibited favorable characteristics without observable adverse effects in experimental animals. This novel fusion protein shows the potential for ameliorating hyperuricemia and related renal complications, highlighting it as a promising drug candidate with substantial market applications.

## 1 Introduction

Increased evidence from clinical studies indicates that hyperuricemia is a high-risk factor for gout, myocardial infarction, chronic renal disease, hypertension, lipid profiles, and cardiovascular diseases [1,2,3,4,5,6,7]. Uric acid is the end product of purine metabolism and the solubility in the blood is poor when the concentration is approximately 6.8 mg/dL [8]. Hyperuricemia arises when the blood uric acid levels exceed this solubility limit emphasizing the necessity for a therapeutic strategy centered on crystal dissolution for effective hyperuricemia management. Conventional acute gout treatment often involves surgery for direct stone removal or crystal discharge, imposing a significant physical burden on patients. For regular hyperuricemia, the existing chemical drugs are generally well tolerated, but there are some unresolved problems with toxicity and side effects [9]. Consequently, it is imperative to develop a safe and effective biological agent that is of pressing importance.

Uricase, often referred to as urate oxidase, can convert uric acid into allantoin, a substance that is highly soluble and easy to defecate [10]. Unlike most animal species, humans and higher apes lack the enzyme uricase [11]. Two nonsense mutations (CGA/AGA → TGA) in the 33rd and 187th amino acid sequences of the human uricase gene alter the codon encoding arginine into a stop codon, leading to premature termination of the coding region, resulting in the loss of functional urate oxidase. This enzyme deficiency results in elevated blood uric acid concentrations in humans. In recent years, a variety of artificial recombinant uricases have been developed and related clinical studies have been done by which a promising application prospect was attained [12,13,14]. However, uricase directly as a medication still has issues with immunogenicity, storage, dissolution, and sustained release; therefore, further advancement is needed [15].

Polyethylene glycol (PEG) has been employed as a pharmaceutical excipient for centuries. PEGylation increases the molecular weight of protein drugs, aiding in avoiding glomerular filtration, reducing immunogenicity and protease degradation, and augmenting solubility and stability while significantly minimizing the release rate. PEG’s low toxicity and superior water solubility have rendered it a preference among pharmacists for its ability to enhance various pharmaceutical dosage forms, particularly in reducing drug half-life and immunogenicity. The homology of microbial uricases to human uricases is modest. Studies on PEGylated microbial uricase conducted internationally during the preclinical research stage were halted as PEGylation did not effectively diminish their immunogenicity. In contrast, PEGylated mammalian uricases have been successfully marketed [15], demonstrating that the immunogenicity of the less immunogenic mammalian uricase can be further reduced through PEGylation.

This study aimed to develop a PEGylated porcine–human recombinant uricase (PEG-PHRU) and evaluate its *in vivo* effectiveness. The pharmacokinetics and pharmacodynamics of PHRU were assessed in Sprague Dawley (SD) rats. Furthermore, the influence of PHRU on body weight, plasma uric acid levels, and renal injury in hyperuricemic rats was also examined. The development of PEG-PHRU presents a promising treatment alternative for gout and related health issues, simultaneously reducing immunogenicity and enhancing treatment efficacy.

## 2 Materials and methods

### 2.1 Animal care, feeding, housing, and enrichment

6–8 weeks old (180–230 g) SD rats were sourced from the Laboratory Animal Research Center of Hubei Province and marked with 1% picric acid. Housing conditions were meticulously controlled; two rats per cage were housed in an individually ventilated environment, maintaining a temperature of 22 ± 1°C, a relative humidity of 40–70%, and a 12-h light/dark cycle. Rats were allowed to drink and eat freely.


**Ethical approval:** The research related to animal use has been complied with all the relevant national regulations and institutional policies for the care and use of animals and has been approved by the Ethics and Research Committee of Hubei Provincial Center for Disease Control and Prevention (No. 201702032). All methods were carried out in alignment with relevant guidelines and regulations. This study was carried out in compliance with the ARRIVE guidelines. The research was performed at a Good Laboratory Practice-compliant facility, fully accredited by the International Association for the Assessment and Accreditation of Laboratory Animal Care.

### 2.2 Anesthesia and euthanasia

All animal operations were performed under isoflurane anesthesia. Animals were executed using an excess infusion of pentobarbital sodium.

### 2.3 Reagents

Allopurinol tablets were procured from Shimao Tianjie Pharmaceutical (Jiangsu) Co., Ltd. Carboxymethylcellulose sodium, boric acid (Guaranteed reagent), disodium tetraborate decahydrate (Analytical Reagent), perchloric acid (Analytical Reagent), and acetic acid (Analytical Reagent) were all acquired from Sinopharm Chemical Reagent Co., Ltd. (Shanghai, China). Potassium oxonate and adenine were sourced from Shanghai Macklin Biochemical Technology Co., Ltd. Yeast extract and 0.9% sodium chloride injection were purchased from Shanghai Chunshi Biotechnology Co., Ltd. (Shanghai, China) and Kelun Pharmaceutical Co., Ltd. (Sichuan, China), respectively. High-performance liquid chromatography (HPLC) grade methanol was obtained from TEDIA Company Inc (USA).

### 2.4 Protein preparation and identification

The PHRU gene was artificially synthesized. *Escherichia coli* containing human and porcine uricase code was provided by Wuhan Haohai Xingkong Biotechnology Co., Ltd. Gene sequences were cloned from pig and human liver tissue using reverse transcription polymerase chain reaction (RT-PCR). The N-terminal sequence of the porcine uricase gene was cloned by RT-PCR, and the C-terminal sequence of the human uricase gene was obtained by artificial synthesis. The N-terminal and C-terminal sequences were ligated into a PHRU chimeric gene. Site-directed mutagenesis was employed to modify the codons at positions 33 and 187 of the uricase amino acid sequence from stop codons to arginine codons (CGA → TGA). The recombinant sequence was inserted into the T vector and transformed into *E. coli* DH5α. The recombinant gene was extracted from the cloned plasmid vector and inserted into the pET28a plasmid vector, creating an expression plasmid that was then transformed into *E. coli* BL21 to generate the expression engineering strain *E. coli* BL21/pET28a-PHRU. The protein was extracted and purified after fermentation of the engineered bacteria. Upon the culture medium reaching an OD value of 1.4, the inducer α-lactose was added to a concentration of 5–6 mmol/L and incubated for 5 h. Bacteria were harvested and disrupted using ultrasonic waves. The expressed protein was purified through an ion exchange column (DEAE Sepharose Fast Flow), hydrophobic column (Phoenyl Sepharose 6 Fast Flow), and gel filtration column (Sephacryl S 300HR). Protein molecular weight was measured by sodium dodecyl sulfate-polyacrylamide gel electrophoresis (SDS-PAGE) [16]. The related synthesis method has been patented (CN102260653B).

### 2.5 PEGylation

A standard injection with an effective activity of 14 U/mL, a purity of 99%, and a molecular weight of 530 kDa are the results of PEGylation done by Wuhan Haohai Xingkong Biotechnology Co., Ltd. The purified PHRU in tetrameric form was dissolved in 0.02 M PBS buffer (pH 7.4) and adjusted to 5–10 mg/mL in 0.1 M carbonate buffer (pH 10.53), and mPEG-SC-10K (Beijing Kaizheng Bioengineering Development Co., Ltd) was added to the solution, with the amount calculated using the formula: mPEG-SC-10K (mg) = (13.5 × PHRU mg)/3.3. The mPEG-SC-10K was gradually added to the enzyme solution and continuously stirred for 30 min, with 1/2 of the total added initially, followed by ¼. The resulting PEG-PHRU was purified using a Sephacryl S-300HR column equilibrated with 0.1 M phosphate buffer (pH 7.4), and samples were collected from the peak. The relative molecular weights of PHRU and PEG-PHRU were determined using size-exclusion (SE)-HPLC-Multi-Angle Laser Scattering-Differential Refractometer, and solubility was evaluated using carbonate buffers across various pH levels. The company provides all pertinent quality control reports.

### 2.6 Degradability analysis

To perform the assay, 150 μL of uric acid solution (1,000 μM) was mixed with PEG-PHRU and incubated at 37°C for 10 min. The reaction was halted by adding 100 μL of 1 M perchloric acid and immediately placed on ice for 5 min. Following centrifugation at 10,000 revolutions per minute (RPM) for 10 min at 4°C, the supernatant was diluted with mobile phase B with the ratio of 1:2 (v:v) before analysis. The uric acid stock solution (2.00 mmol/L) was prepared by dissolving the compound in methanol, and a series of standard uric acid solutions were obtained by further diluting the stock solution with substrate buffer. The uric acid working solution was diluted with mobile phase B having the ratio of 1:2 (v:v) before HPLC analysis.

Analysis was performed on a Shimadzu LC-2010A HPLC system that was equipped with a UV detector, a quaternary pump, a column temperature compartment, and an autosampler. Symmetry C18 column (4.6 mm × 250 mm) (WATERS, USA) of 5 μm particle size was utilized at 30°C. Mobile phase C was methanol, while mobile phase B comprised water with 0.1% acetic acid and methanol (9:1, v-v). A maintained gradient flow at a flow rate of 1.2 mL/min was used. The applied gradient elution was as follows: 5% C from 0 to 0.8 min, 5–10% C from 0.8 to 1.8 min, 10% C from 1.8 to 2.8 min, 10–15% C from 2.8 to 3.5 min, 15–5% C from 3.5 to 3.6 min, and 5% C from 3.6 to 5.5 min. A 20 μL sample was injected each time, and UV detection was performed at a wavelength of 288 nm.

The PEG-PHRU concentration in plasma was calculated indirectly by the uric acid reduction value. The residual uric acid post-PEG-PHRU reaction in the plasma sample with uric acid as the substrate was extrapolated based on the standard calibration curve, constructed by plotting uric acid standards versus peak area. This relationship facilitated the determination of PEG-PHRU concentration in plasma samples.

### 2.7 Pharmacodynamic model

This study included a total of 80 male rats, weighing between 180.1 and 239.4 g, randomly divided into two groups: a blank control group (10 rats) and a hyperuricemia model group (70 rats). Hyperuricemia was induced through intraperitoneal injection of reagents for 12 consecutive days (15 mg/kg body weight; yeast extract 1.00 g/mL, adenine 13.33 mg/mL, potassium chlorate 2.00 mg/mL). Blood samples were collected from the jugular vein 1 h after administration of the molding agent on the 12th day for plasma uric acid concentration analysis. Based on plasma uric acid levels, 50 rats were selected and divided into five groups: model group, allopurinol group, low-dose group, medium-dose group, and high-dose group, each containing 10 rats. To maintain high plasma uric acid levels, the agent was administrated until the 23rd day. The rats in the allopurinol group were administered allopurinol suspension (prepared by mixing the powder of two allopurinol tablets into a 0.5% carboxymethylcellulose sodium suspension) once a day at a dose of 20 mg/kg of allopurinol for 11 days starting the day after grouping. On the 14th day after hyperuricemia modeling, the low, medium, and high dose groups were tail vein injected with the PEG-PHRU 0.42, 0.84, and 1.68 mg/kg, respectively, while the solvent control group was injected with an equal volume of 0.9% sodium chloride injection. To evaluate potential changes in body weight, pre- and post-administration measurements were conducted. The drug dosage for rats was determined using the Meeh-Rubner formula as a reference standard, taking into account body surface area [17]. Following conversion, the allopurinol dose was established at 20 mg/kg, aligning with the minimum toxic dose reported in previous studies [18].

### 2.8 Plasma test

After administration, the plasma uric acid levels of each group were analyzed using 0.1 ml of blood collected from the jugular vein at various time points (2 h and 1, 2, 3, 4, 5, 6, 7, and 9 days). The plasma uric acid levels were measured using a commercial kit (D799286, Nanjing Sangon, China).

### 2.9 Histology

The tissue samples were fixed in 10% formalin for 48 h, followed by dehydration and embedding in paraffin. Microscopic examination was conducted after sectioning at 5 μm and staining with H&E (BL700A, Biosharp, Hefei, China). The protocols are as previously stated [19,20]. For further details on tissue processing and staining methods, we refer readers to the relevant references [21–25].

### 2.10 Pharmacokinetic model

Twenty-four rats, weighing between 180.6 and 232.8 g, were selected randomly, ensuring an equal gender distribution across the following three groups: a low-dose group (0.42 mg/kg), a medium-dose group (0.84 mg/kg), and a high-dose group (1.68 mg/kg). We conducted single-dose pharmacokinetic studies of PEG-PHRU administered via tail vein injection. Blood samples were collected from the jugular vein using heparin sodium as an anticoagulant at different time points (0, 0.167, 2, 4, 8, 12, 16, 24, 36, 48, 72, 96, 120, 144, 192, 240, 336, and 384 h) post-administration. 0.5 mL blood samples were used for quantification of plasma PEG-PHRU concentrations. Plasma was separated by centrifugation at 3000 RPM for 10 min.

### 2.11 Pharmacokinetic analysis

Pharmacokinetic parameters were determined using a non-compartmental model with uniform weighting for intravenous infusion dosing, integrated into the WinNonlin software (Version 7.0, Pharsight Corporation, Cary, NC, USA). The calculated parameters encompassed the half-life (*t*_1/2_), maximum plasma concentration (*C*_max_), area under the plasma concentration–time curve extrapolated to infinity (area under curve, AUC_0−*t*_), apparent volume of distribution (V_d_), and apparent clearance (CL) for PEG-PHRU.

### 2.12 Statistical analysis

Data were analyzed using SPSS 21.0 (SPSS Inc., Chicago, IL, USA) and are presented as mean ± standard deviation. Statistical significance was determined using one-way analysis of variance (ANOVA) or repeated measure ANOVA. The Student’s *t*-test compared differences between the two groups, considering a *p*-value of <0.05 as indicative of a significant difference.

## 3 Results

### 3.1 Characterization, expression, and identification of PHRU

**Figure 1:**
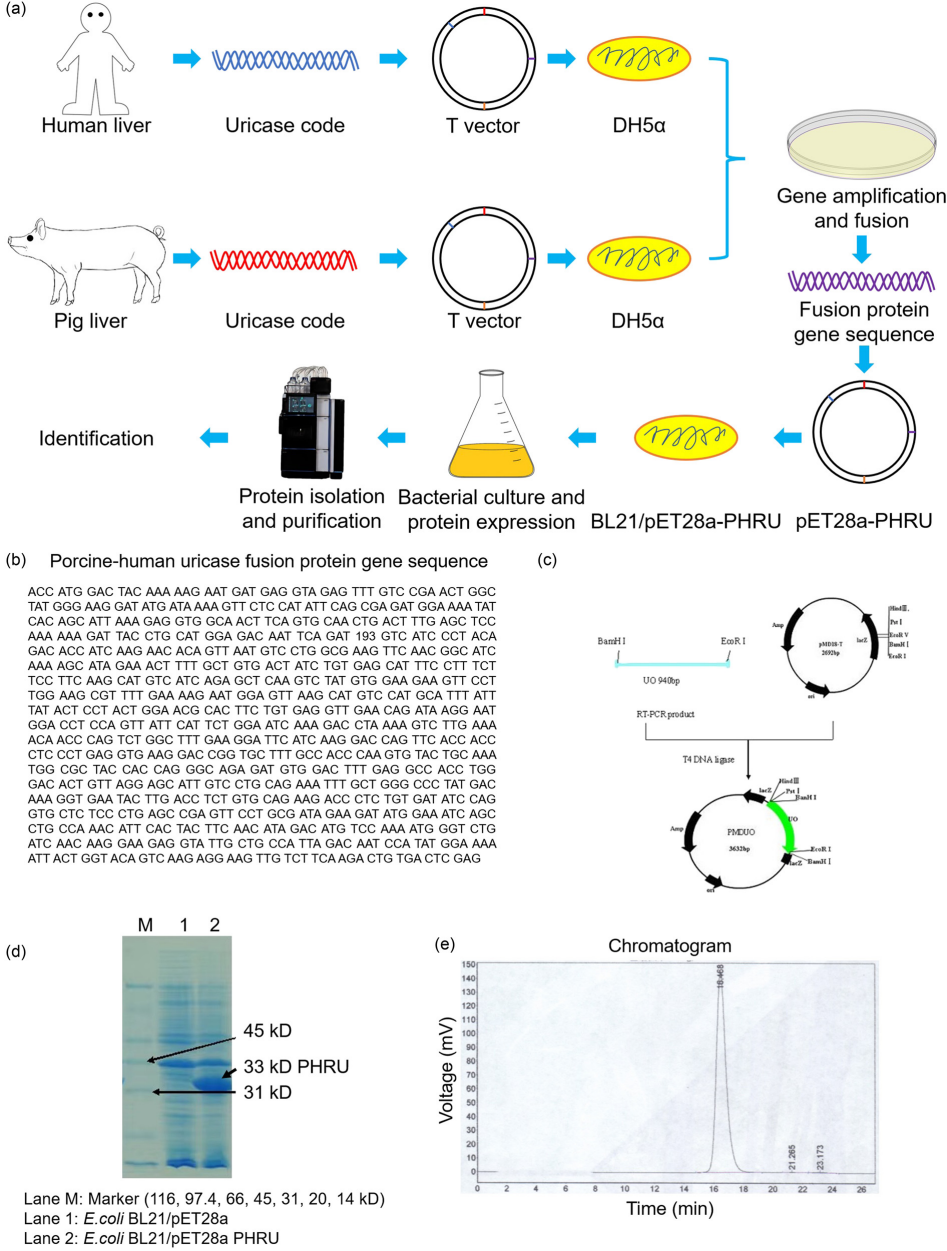
Synthesis method of PHRU. (a) Schematic illustration of the synthesis pathway. (b) Gene sequence of the fusion protein. (c) Schematic diagram of the vector. (d) Representative graph of protein expression identified by SDS-PAGE. PHRU is revealed in the 33 kD band. (e) Graph of HPLC protein purification results.

The experimental procedure entailed introducing the complete PHRU gene into plasmid pET28a and expressing it in *E. coli* BL21, resulting in the formation of BL21/pET28a-PHRU (Figure 1a–c). Following incubation, successful expression of PHRU was confirmed by SDS-PAGE, which demonstrated a molecular weight of approximately 33 kDa (Figure 1d). HPLC purification yielded a highly pure (99.6078%) recovery peak of PHRU at 16–18 min (Figure 1e). Size-exclusion HPLC (SE-HPLC) analysis revealed that the purified PHRU existed as tetramers, with a relative molecular weight of 131.3 kDa. These results underscore the efficacy of the experimental design in the expression and purification of PHRU.

### 3.2 Characterization and degradability test of PEG-PHRU

**Figure 2:**
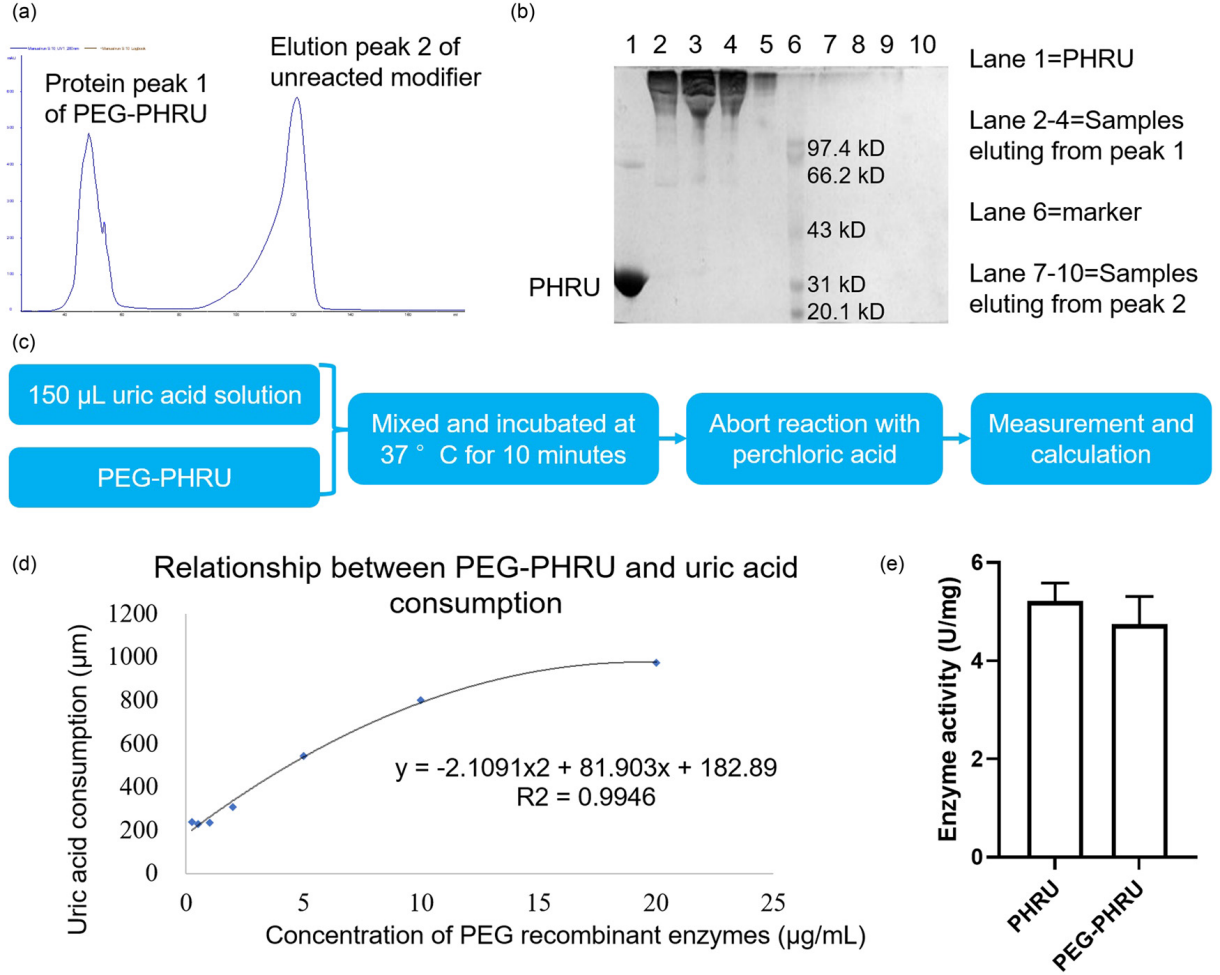
Characterization and degradability test of PEG-PHRU. (a) Plot of elution peak at 280 nm for separation and purification by gel chromatography. (b) SDS-PAGE electrophoresis results were separated and purified by gel chromatography. (c) The third-party company successfully PEGylated the PHRU. The results show that PEG has been added to PHRU. (b) Experimental procedure for degradability testing. (d) PEG-PHRU exhibited a dose-dependent ability to reduce uric acid levels. (e) Results of enzyme activity and residual activity before and after PEGylation. Tested at 8 mg/mL, pH 9.16.

Following the synthesis of PHRU, the compound was sent to a third-party company for PEGylation. The synthesis of PEG-PHRU was subsequently evaluated. As depicted in Figure 2a and b, HPLC and SDS-PAGE results showed different protein peaks and protein accumulation bands, respectively, confirming the successful PEGylation process. This PEG-PHRU formulation was then utilized for further investigations.

To initially assess the degradation potential of PEG-PHRU toward uric acid, we conducted experiments to explore the relationship between PEG-PHRU and uric acid concentration (Figure 2c). As anticipated, PEG-PHRU demonstrated a dose-dependent ability to reduce uric acid levels (Figure 2d). Notably, it is worth mentioning that the uric acid degradation rate began to plateau once the administered drug concentration exceeded 15 μg/mL. To observe the effect of PEGylation on the enzyme activity of PHRU, we did the same test. The results revealed that PEGylation reduced the PHRU enzyme activity with a residual enzyme activity of about 91% (Figure 2e).

### 3.3 PEG-PHRU reduces hyperuricemia without affecting body weight

**Figure 3:**
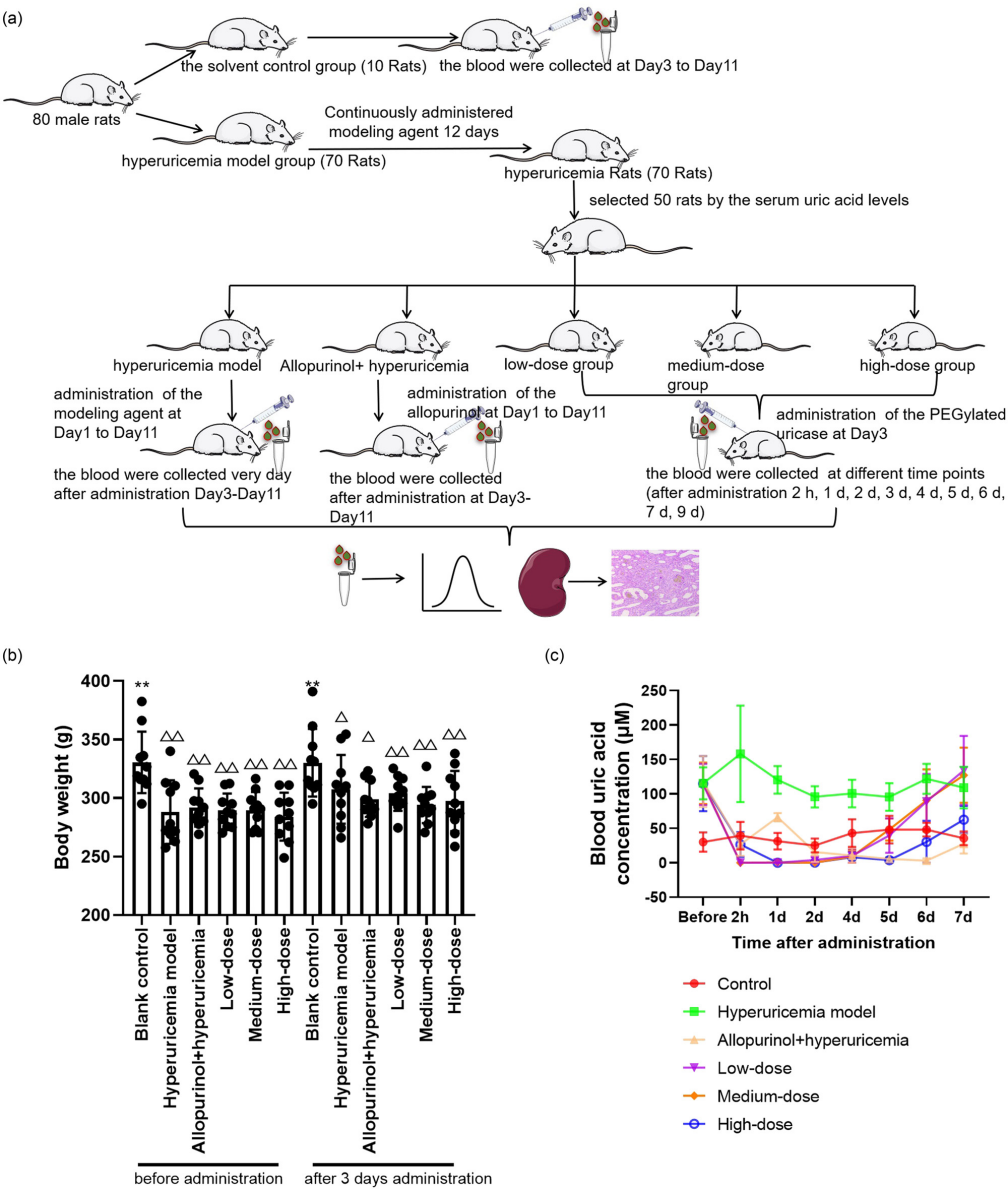
PEG-PHRU improves SD rat’s hyperuric acidemia. (a) Schematic diagram of the experimental process. (b) The effect of PEG-PHRU on the body weight of rats. *N* = 8–10. (c) PEG-PHRU reduces plasma uric acid in a dose-dependent manner. *N* = 8–10. *, comparison with the model group. △, comparison with the control group. *p ≤ 0.05; **p ≤ 0.01.

The efficacy of PEG-PHRU injection for treating hyperuricemia was assessed in hyperuricemic rats (Figure 3a). Initially, the impact of intravenous administration of the drug on rat weight was measured, and a significant decrease in body weight was observed in all groups (Figure 3b). This weight loss was attributable to the use of modeling agents for inducing animal models of hyperuricemia. However, after 3 days of drug administration, there was no significant difference in the body weight of animals in different groups, indicating that the various drug doses did not affect the rat weight. Moreover, following the administration of low, medium, and high doses of PEG-PHRU injection in rat tails with hyperuricemia, a significant reduction in plasma uric acid levels was observed. The plasma uric acid levels remained controlled for 5 days with low and medium dosages and for a minimum of 7 days with the high dose (Figure 3c). These findings signify that PEG-PHRU injection shows promising results in treating hyperuricemia.

### 3.4 PEG-PHRU injection reduces urate deposition and attenuates kidney injuries

**Figure 4:**
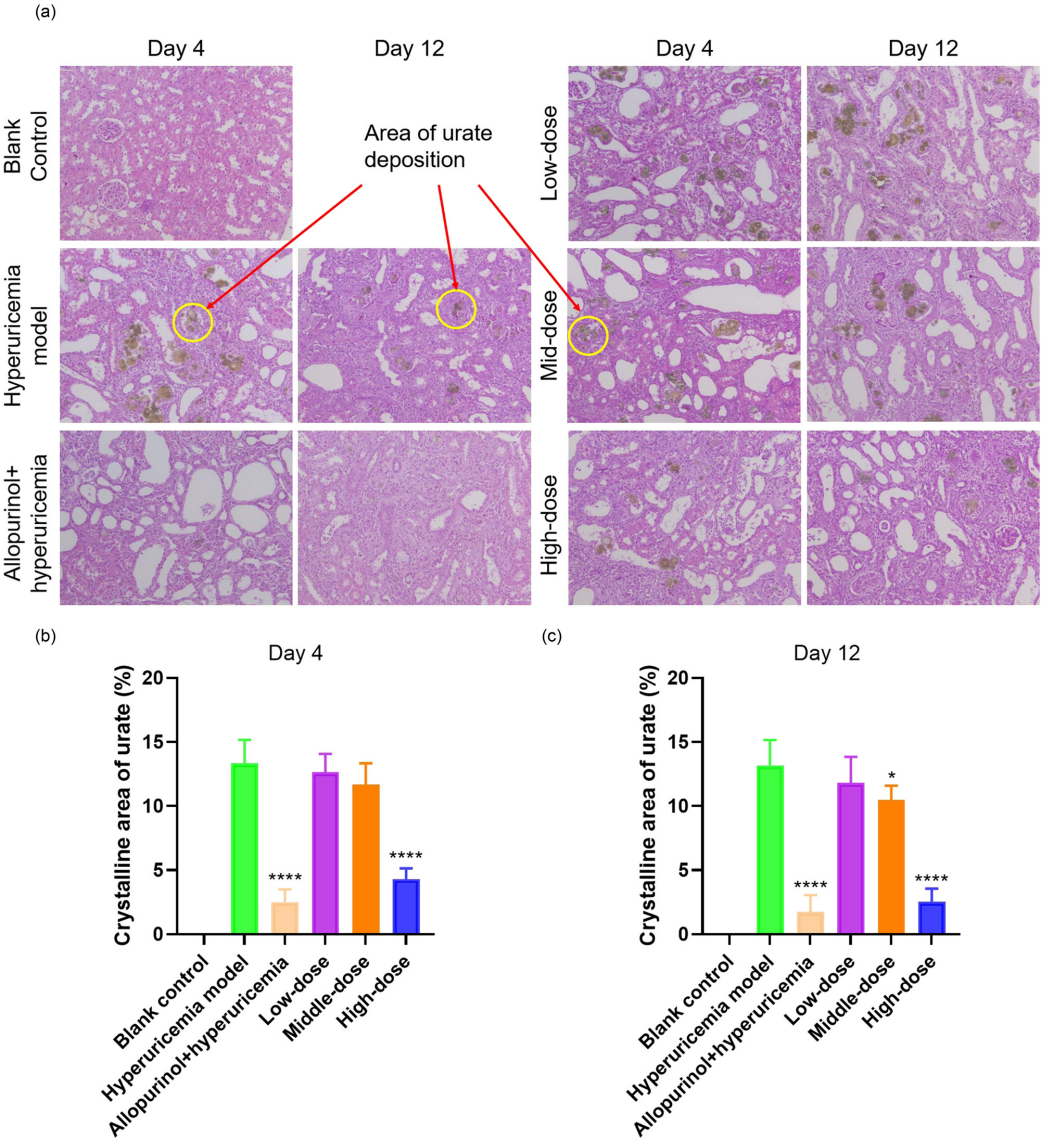
PEG-PHRU injection reduces urate deposition and attenuates kidney injuries in a dose-dependent manner in hyperuricemic rats. (a) Representative images of kidney sections with hematoxylin–eosin staining (H&E) staining. (b and c) Statistical results of urate crystallization in the kidneys. * represents a comparison with the model group. ****, *p* ≤ 0.0001.

The accumulation of urates in the kidneys increases the probability of kidney stones. To further evaluate the therapeutic efficacy of PEG-PHRU on hyperuricemic rats, we utilized tissue staining to analyze and compare the histological changes in kidneys (Figure 4a–c). Rats had intact membranes, clearly shaped renal corpuscles, and proximal and distal convoluted tubules before modeling, serving as a normal control for tissue samples. On the twelfth day following modeling, rats experienced significant increases in renal volume, accompanied by dilated renal tubes, renal tube epithelial cell degeneration, necrosis, shedding, and a large quantity of brown crystallization in the tube cavity. This was coupled with interstitial fibrosis, inflammatory cell infiltration, and occasional foreign giant cells, some of which contained brown-yellow crystallization. After administering PEG-PHRU, renal lesions in rats remained consistent with the model group on the fourth day of treatment, with no reduction observed in renal tubule epithelial cell lesions except for a slight reduction in one rat in the high-dose group. In subsequent experiments, continued administration of the drug resulted in pathology tests revealing that animals in the high-dose group exhibited a significant reduction in brown crystallization in renal tissue, while other lesions were comparable to those in the model group.

Furthermore, we conducted staining tests on additional tissues, including the liver and spleen, but no significant pathological differences were observed (data not shown). This observation aligns with the known hyperuricemia impacts primarily on the joints, kidneys, and cardiovascular system, exerting limited effects on other organs. Consequently, no evident lesions were detected in other tissues throughout the study period.

### 3.5 Pharmacokinetics

**Figure 5:**
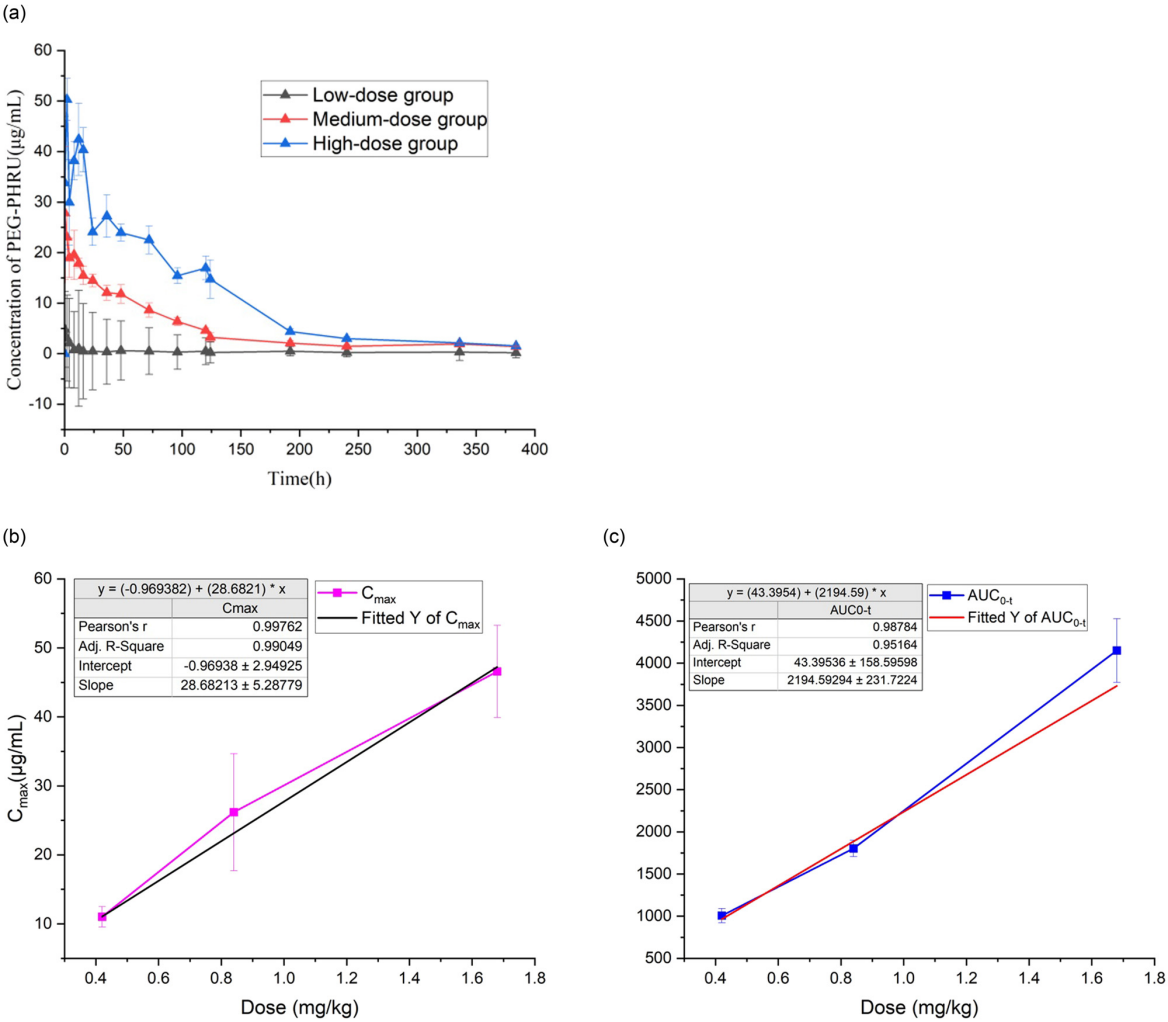
Pharmacokinetic parameters of three groups of rats after intravenous injection of three different doses of PEG-PHRU, respectively. (a) Twenty-four rats were divided into three groups and injected intravenously with low, medium, and high doses of PEG-PHRU. This graph shows the relationship between the mean concentration of PEG-PHRU in plasma and time. (b) The linear relationship between *C*_max_ and drug dose after injection. (c) The linear relationship between AUC_0–384 h_ and drug dose post-injection.

To investigate the pharmacokinetics of PEG-PHRU in rats, we conducted a study involving 24 rats. They were divided into three groups, and each group received a different dosage of PEG-PHRU via tail vein injection: low, medium, and high doses. Figure 5a illustrates the average plasma concentration of PEG-PHRU over time. Notably, all drug concentrations in plasma exhibited a declining trend over time. Among these, the high-dose group displayed a half-life of approximately 48 h. After 240 h, the residual drug concentration in plasma was nearly negligible across all three groups. Additionally, these data facilitated the determination of several PEG-PHRU pharmacokinetic parameters, including the half-life (*t*_1/2_), *C*_max_, AUC_0−*t*_, Vd, and CL. The calculated values for these parameters are summarized in Table 1.

**Table 1:** Pharmacokinetic parameters (mean ± standard deviation) after intravenous injection of PEG-PHRU in mice

Group	Low-dose group	Medium-dose group	High-dose group
Dose (mg/kg)	0.42	0.84	1.68
*t*_1/2_ (h)	113.3 ± 19.0	96.8 ± 8.9	121.5 ± 39.3
*C*_max_ (μg/mL)	11.0 ± 1.5	26.2 ± 8.5	46.6 ± 6.7
AUC_0–384_ (h μg/mL)	1,007 ± 84	1,803 ± 97	4,151 ± 378
AUC_0−∞_ (h μg/mL)	1,173 ± 125	1,999 ± 107	4,429 ± 420
*V*_d_ (mL/kg)	62.7 ± 6.7	59.9 ± 4.8	0.383 ± 0.040
CL (mL/h/kg)	0.362 ± 0.038	0.422 ± 0.023	46.6 ± 5.8

*N* = 8 in each group.

Furthermore, a linear regression analysis was conducted to determine the relationship between *C*_max_ and AUC_0−*t*_ with respect to the administered dose (Figure 5b and c). The calculated correlation coefficients (*r* ^2^) for the linear regressions were approximately 0.9 in all cases, highlighting a positive correlation between *C*_max_, AUC_0−*t*_, and the administered dose within the dose range of 0.420–1.68 mg/kg. Consequently, the pharmacokinetics of PEG-PHRU in this dose range exhibit linear characteristics.

## 4 Discussion

The rising global incidence of hyperuricemia underscores the imperative demand for the development of more potent medications for patients with hyperuricemia. Although a few recombinant uricase drugs have been approved for marketing, their practical use is hampered by persistent immunogenicity issues. This study presents a comprehensive preclinical development plan, showcasing promising outcomes, and holding significant potential to transition into clinical trials, thereby playing a pivotal role in treating hyperuricemia and its associated complications.

Currently, uricase injections are an effective treatment for hyperuricemia and those unresponsive to conventional treatments. However, due to the presence of two fatal mutations in the human uricase gene [11], individuals are unable to efficiently degrade uric acid. To develop a uricase preparation suitable for human use, we replaced the non-functional part of the human gene with a pig uricase-derived gene, exhibiting enhanced uricase activity and resembling the human gene. This resulted in the PHRU chimeric gene, which can be used to produce PHRU for clinical use.

Clinical observations have shown that maintaining the efficacy of protein drugs after multiple administrations can be challenging, as exogenous proteins act as natural antigens, prompting the body to produce antibodies post-injection. This can affect subsequent treatment efficacy and cause allergic reactions in severe cases. PEGylation has been identified as an effective solution to this problem by reducing immunogenicity and prolonging half-life [26]. PEG is an uncharged linear hydrophilic molecule that covalently binds to non-essential groups of proteins to form a spatial conformational barrier, preventing the induction of antibodies by epitopes of foreign proteins. PEGylation has been widely recognized in clinical applications, with clinical studies targeting PEGylated uricase confirming its efficacy in significantly reducing immune rejection [27]. However, some clinical studies have observed that PEGylated uricase injection can cause the body to produce antibodies against PEG, but not against uricase [12,28]. The reason for this result could be the different sources of uricase and the different production protocols of PEG. In this study, the fusion protein was modified by PEGylation to reduce the antigenicity of PHRU and preserve its enzymatic activity. Subsequent animal experiments confirmed the efficacy and biosafety of the new drug, observing no significant immune rejection, thereby indicating the success of the drug design and optimization.

Hyperuricemia is commonly associated with complications such as gout and kidney stones, attributed to the accumulation of urate crystals in these areas. While surgical interventions are routinely employed, *in vivo* degradation emerges as an alternative solution. Our study demonstrates the efficacy of PEG-PHRU in lowering blood uric acid levels and mitigating urate crystal accumulation in the kidneys. Notably, no significant impact on body weight or physiological functions of rats was observed, indicating the safety of PEG-PHRU. Our findings provide preliminary evidence supporting the efficacy and safety of PEG-PHRU. Furthermore, the pharmacokinetic analysis revealed a half-life exceeding 90 h, suggesting that frequent injections can be avoided. Additionally, the linear pharmacokinetic characteristics within the dose range indicate the controllability of PEG-PHRU’s dose and effect. These favorable attributes highlight PEG-PHRU’s potential for clinical applications.

Currently, several recombinant uricase enzymes are used in therapeutic settings. Rasburicase, a recombinant aflatoxin oxidase, is commonly used to manage blood uric acid levels in patients, particularly those with tumor lysis syndrome [29,30]. However, its short half-life of approximately 19 h and strong antigenic nature may result in reduced efficacy and antibody production with prolonged use [30,31,32,33]. Therefore, caution should be exercised when administering Rasburicase in patients who are sensitive to fluctuations in serum uric acid levels and who are prone to allergies. Moreover, Pegloticase, a PEGylated porcine-like uricase produced by *E. coli* [15], has a longer half-life and higher bioavailability than Rasburicase [34]. However, its prolonged use may lead to poor therapeutic efficacy due to the production of high titers of Pegloticase antibodies [35,36,37,38]. In fact, side effects related to hypersensitivity reactions are still reported more frequently for both drugs, constraining their application scope. Recent attempts to use uricase in combination with immunosuppressants in practice have met with some success, but this has inadvertently introduced new immune-related problems. Compared to these two drugs, PEG-PHRU in this study may have lower theoretical antigenicity than the aforementioned drugs. PEG-PHRU, the resulting product after extending the half-life of the drug with PEG, had a half-life of over 90 h. However, to establish its antigenicity over a longer period, more research is necessary. Presently, investigations to juxtapose the efficacy and side effects of these uricase-like drugs are underway.

There are some limitations in this study. First, it is important to acknowledge that our study only provided a preliminary exploration of the short-term safety of PEG-PHRU. A comprehensive evaluation of the drug’s safety and potential gender differences in drug effects remains unexplored. Second, we did not assess the levels of uric acid-related metabolites beyond the scope of this study. Third, protein structure and expression optimization, as well as short-term and long-term assessment of immune rejection following drug injection, were not analyzed. These aspects warrant consideration in forthcoming research to attain a more comprehensive understanding of PEG-PHRU. Last, it is noteworthy that our study mandated intravenous injection for the effective administration of PEG-PHRU. However, recent advancements propose the utilization of engineered exosomes for oral and targeted drug delivery, which can minimize the trauma associated with drug administration and enhance delivery specificity [39,40]. This pathway represents a promising focus for future research endeavors. A comprehensive examination of this drug’s effects in ensuing studies should traverse a diverse spectrum of areas.

## 5 Conclusions

In conclusion, this study introduces a novel synthesis method for the production of PEG-PHRU and preliminarily verifies its efficacy and safety through preclinical studies. Notably, PEG-PHRU demonstrated dose-dependent efficacy in treating hyperuricemia and its complications. These findings provide a promising therapeutic strategy for the clinical treatment of uric acid degradation.
